# Illinois State Veterinary Medical Association, and Iowa State Veterinary Medical Association

**Published:** 1891-01

**Authors:** 


### ILLINOIS STATE VETERINARY MEDICAL ASSOCIATION, AND IOWA STATE VETERINARY MEDICAL ASSOCIATION.

We regret extremely that we are unable to furnish our readers with further information in regard to the meetings of the Illinois State Veterinary Medical Association, and the Iowa State Veterinary Medical Association.

Our first communications to the officers of these associations remained unnoticed, and when we wrote later, stating that we were holding our issue for their reports, we received no reply. We have since been notified that both Associations "by vote give exclusive use of its papers to '*The Review*.'

We are sorry that our colleagues of Illinois and Iowa should seem to regard their mental productions, and any reports of their discoveries and experience in veterinary medicine, in the nature of a patent medicine, whose contents cannot be given to the public. No science, and less so veterinary medicine, is extensive enough to neglect any opportunity of advancing it, and we assure our readers that all matters of *value* will be placed before them *when it is obtainable*.

